# Introduction to the special topic on nanomanufacturing

**DOI:** 10.1038/micronano.2017.79

**Published:** 2017-09-25

**Authors:** Eric M. Yeatman, Hannah M. Gramling, Evelyn N. Wang

**Affiliations:** 1 Fellow of the Royal Academy of Engineering and Professor, Electrical and Electronic Engineering, Imperial College London, South Kensington Campus, London SW7 2AZ, UK; 2 Mechanical Engineering, University of California Berkeley, Berkeley, CA 94720, USA; 3 Professor, Mechanical Engineering, Massachusetts Institute of Technology, 77 Massachusetts Avenue, Cambridge, MA 02139, USA

The semiconductor industry introduced sub-100-nanometer technology to consumers more than a decade ago, in the early 2000s. The field of ‘nanotechnology’ has since expanded rapidly, drawing on methods and materials from integrated electronics while adding many others, such as quantum dots, nanowires and self-assembly, and targeting applications in optoelectronics, biomedical devices, sensors, fluidics and many other areas. The isolation of monolayer graphene in 2004, in particular, initiated a massive worldwide research effort in 2D materials. While the volume of papers published in the realm of nanotechnology continues to balloon year-on-year, surprisingly few of the technologies—which are frequently deemed to hold particular promise—have found their way to commercialization.

Two key roadblocks are responsible: the lack of a driving technological pull (the so-called ‘killer app’) for many of the nanotechnologies in question, and the ability to produce the nanotechnologies at scale.

This Special Topic addresses the second of these obstacles, by taking stock of the state of ‘nanomanufacturing’, examining a commercial success in the field, and introducing processes that can fill in the gaps between one-off demonstrations and mass production.

Despite the use of the term ‘nanomanufacturing’ in a number of previous conferences and journals, we found that few papers truly address manufacturing issues related to nanotechnology. In many cases the technologies described are not really at the nanoscale, or the papers present prototype fabrication methods, rather than manufacturing techniques. Nanomanufacturing—the development of scalable, high-yield processes for the commercial production of materials, structures and devices at the nanoscale—will be fundamental to realizing the promise of nanotechnologies.

This Special Topic is being introduced with an initial set of six papers, with up to four more to be added in the coming months. All the papers in this issue were invited. They cover a diverse set of manufacturing technologies. S.V. Sreenivasan presents the development of Jet and Flash Imprint Lithography (J-FIL) from initial research to a commercial system. J-FIL is an alternative to the optical methods of integrated circuit manufacture for nanoscale patterning at high production volumes and over large areas. This paper provides a case study of the evolution of a nanomanufacturing method that contains valuable lessons for techniques currently undergoing this evolution.

In ‘Nanoimprint for producing layered nanostructures’, Liang and colleagues explore the manufacturing applicability of their process for producing patterned nanostructures of molybdenum disulfide (MoS_2_) by probing scaling limits. They show that there is an aspect ratio limit beyond which the quality of the transferred material is significantly degraded, and estimate the maximum lateral dimension for producing undamaged few-layer material. In the process, they uncover a unique behavior of shear-exfoliated material that may lend itself to device exploitation. While direct growth of atomic layers is the subject of intense research effort, the deployment of two-dimensional (2D) materials such as MoS_2_ will span a range of applications; techniques such as that demonstrated by Liang *et al.* can speed the introduction of 2D materials into mass device fabrication for thickness-agnostic applications.

Volume manufacturing of high aspect ratio (HAR) micro-structures is well established, but its extension to nanostructures, and to the combination of nano- and micro-structures, presents additional challenges. This is the topic addressed in ‘High-aspect-ratio nanoimprint process chains’ by Schift and colleagues. They investigate and compare several methods for production of both HAR masters and the final structures, with a focus on manufacture of photonic devices. New techniques are demonstrated, particularly to enable the micro- and nano- combination, with potential for high-throughput, low-cost manufacturing.

The use of nanostructured materials in energy storage is a large and growing research area. Pauzauskie *et al.*, in ‘Rapid synthesis of transition metal dichalcogenide-carbon aerogel composites for supercapacitor electrodes’, investigate the manufacture of transition metal dichalcogenides (TMDs) for supercapacitors. They demonstrate a technique for nanomanufacturing of composites of TMDs and carbon aerogels, obtaining the high energy of the former along with the stability and high surface area of the latter in a scalable, low-cost process.

Self-assembly is another promising route to manufacturing nanoscale features over large areas at low cost. Bhaskaran and colleagues, in ‘Nanoparticle assembly enabled by EHD-printed monolayers’, demonstrate such a route using the combination of self-assembly and electrohydrodynamic jet printing. In addition to the manufacturing method itself, they demonstrate how dual-harmonic Kelvin probe microscopy can be used for metrology of the patterned structures.

Nanomanufacturing in most cases is likely to involve a combination of process steps, and so computer-aided design and manufacturing (CAD/CAM) tools will be essential. In ‘CAD/CAM for scalable nanomanufacturing: a network-based system for hybrid 3D printing’, Ahn and colleagues present the key attributes required for a nanomanufacturing CAD/CAM system. They then describe a prototype of such a system and its capabilities, and outline the further developments needed for a comprehensive nanomanufacturing application.

By emphasizing the many engineering challenges of scalable production of nanoscale features, we hope the Special Topic will achieve three goals: that it will inspire more work, and further publications, on nanomanufacturing specifically; that it will encourage researchers to discuss issues of manufacturability in their own work; and that it will illuminate a road map for researchers addressing challenges in nanomanufacturing by surveying the route of tackling these issues, from conception, demonstration and characterization of new techniques in a laboratory setting to translation via commercialization and industry partnerships.

